# Right Pulmonary Artery Bypass After Prior Left Pneumonectomy for Pulmonary Artery Sarcoma

**DOI:** 10.1016/j.atssr.2025.11.023

**Published:** 2025-12-17

**Authors:** Geraud Galvaing, Mehdi Farhat, Jean-Baptiste Chadeyras, Nicolas d’Ostrevy, Marc Filaire

**Affiliations:** 1 Department of Thoracic and Endocrine Surgery, Jean Perrin Comprehensive Cancer Center, Clermont-Ferrand, France; 2 Department of Cardiac Surgery, Clermont-Ferrand University Hospital, Clermont-Ferrand, France

## Abstract

Primary pulmonary artery sarcoma is a rare and aggressive malignant neoplasm with poor prognosis and frequent local recurrence. Complete resection remains the treatment of choice, but repeated surgery is rarely described, especially after a pneumonectomy. Repeated radical resection of recurrent pulmonary artery sarcoma with prosthetic reconstruction is feasible and can be performed without additional lung loss. Three-dimensional modeling is a valuable adjunct for preoperative planning in complex pulmonary vascular surgery and may help preserve pulmonary function in selected patients.

Primary pulmonary artery sarcoma (PAS) is an uncommon malignant tumor originating from the intimal layer of the pulmonary artery, with fewer than 500 cases reported in the literature.^1^^,^^2^ Prognosis is poor, with median survival of 8 to 36 months after surgery,^1^ and local recurrence is frequent. Complete surgical resection remains the mainstay of treatment and is associated with the best survival outcomes.^2^^,^^3^ Repeated surgery for recurrence is rarely described and technically demanding, particularly in aiming to avoid lung parenchymal sacrifice. We present the case of a young patient with recurrent PAS successfully treated with repeated complete resection and prosthetic reconstruction, aided by 3-dimensional (3D) surgical planning.

A 35-year-old man presented with hemoptysis. He had no relevant past medical history. Chest computed tomography (CT) demonstrated a left pulmonary mass with contiguous invasion of the left pulmonary artery. CT-guided biopsy confirmed intimal PAS. After multidisciplinary tumor board discussion, neoadjuvant chemotherapy was administered, followed by left pneumonectomy in September 2021. The left pneumonectomy was performed through a left lateral thoracotomy in a standard fashion. The postoperative course was uneventful, and pathologic examination confirmed a 5-cm PAS with negative margins. Surveillance CT scans remained normal until March 2025, when imaging revealed a right pulmonary artery mass just distal to the truncus anterior, consistent with local recurrence (Figure 1A).

**Figure 1:**
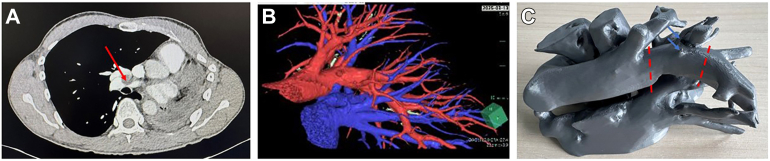
Preoperative planning of the surgery. (A) Preoperative computed tomography scan; the arrow depicts the sarcoma in the right pulmonary artery. (B) Three-dimensional reconstruction of the patient’s pulmonary artery. (C) Three-dimensional printed model derived from the reconstruction; the dashed lines depict the planned resection margins, and the arrows indicate the 2 A2 branches that had to be sacrificed.

At the age of 39 years, the patient was reassessed by the surgical team. Preoperative planning used CT-based 3D reconstruction and a life-size 3D printed model, which demonstrated the feasibility of complete resection without parenchymal resection (Figure 1B). Through a median sternotomy and femoral arteriovenous cardiopulmonary bypass, the branches of the right superior pulmonary vein were dissected and retracted inferiorly while the truncus anterior was mobilized superiorly. The pulmonary artery was opened, and the tumor was excised en bloc with only 2 small A2 branches sacrificed (Figure 1C). A ringed 15-mm polytetrafluoroethylene interposition graft was placed (Figure 2), with end-to-end anastomoses using 4-0 Prolene (Video). Transient atrial fibrillation developed postoperatively, which resolved medically. He was discharged on postoperative day 6 on apixaban and resumed work 6 weeks later. Final histopathologic examination confirmed a 3-cm intimal sarcoma with negative margins. At 3-month follow-up, CT imaging showed complete graft patency and no evidence of recurrence (Figure 2C).

**Figure 2:**
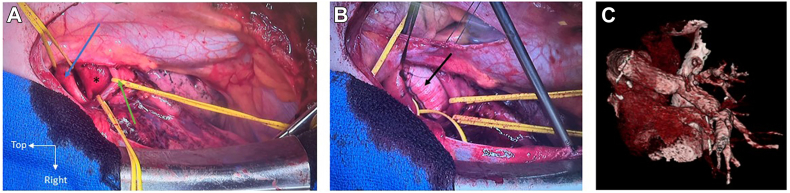
Intraoperative and postoperative views. (A) Intraoperative view showing the truncus anterior (blue arrow), the right superior pulmonary vein (green arrow), and the pulmonary artery trunk (star). (B) Intraoperative view showing the graft after completion of both anastomoses (arrow). (C) Posterior view of the computed tomography scan performed 3 months after surgery, confirming graft patency.

## Comment

PAS often mimics chronic thromboembolic disease, delaying diagnosis.^3^, ^4^, ^5^ Complete surgical resection with negative margins is the treatment of choice and improves survival compared with palliative therapy.^2^^,^^3^ However, recurrence is common, particularly after limited resections.^1^ Repeated surgery is rarely reported because of technical complexity and patient comorbidities.^3^ Peripheral cannulation for cardiopulmonary bypass was selected owing to leftward displacement of the heart and mediastinum, which made central cannulation technically demanding. This strategy also prevented interference of the cannulas while the anastomoses were being performed. The leftward mediastinal shift, however, provided convenient exposure of the extrapericardial segment of the right pulmonary artery, lying directly beneath the sternotomy.

Our case is notable for delayed recurrence after pneumonectomy, successful repeated complete resection without further parenchymal loss, and use of 3D printing for preoperative planning. Similar to other reports, 3D models improved spatial understanding, allowed precise identification of uninvolved arterial segments, and facilitated safe anastomosis planning. This approach preserved right lung function, avoiding postoperative pulmonary hypertension.^1^

Whereas long-term prognosis remains guarded, aggressive surgical management in selected patients may extend survival.^3^ The optimal role of adjuvant therapy is undefined; chemotherapy and radiotherapy may be considered for locally advanced or incompletely resected disease.^1^^,^^4^

Repeated radical resection of recurrent PAS with prosthetic reconstruction is feasible and can be performed without additional parenchymal loss. In complex pulmonary vascular surgery, 3D modeling is a valuable adjunct for operative planning.
